# Microbiome of the Black-Lipped Pearl Oyster *Pinctada margaritifera*, a Multi-Tissue Description With Functional Profiling

**DOI:** 10.3389/fmicb.2019.01548

**Published:** 2019-07-05

**Authors:** Caroline Eve Dubé, Chin-Long Ky, Serge Planes

**Affiliations:** ^1^PSL Research University: EPHE-UPVD-CNRS, USR 3278 CRIOBE, Université de Perpignan, Perpignan, France; ^2^Laboratoire d’Excellence “CORAIL”, Mo’orea, French Polynesia; ^3^Ifremer, UMR 241, Centre du Pacifique, Tahiti, French Polynesia; ^4^Ifremer, UMR 5244 Interactions Hôtes Pathogènes Environnements, Université de Montpellier, Montpellier, France

**Keywords:** pearl oyster, microbiome, tissue-specific bacterial communities, 16S rRNA gene sequencing, functional profiling prediction

## Abstract

Elucidating the role of prokaryotic symbionts in mediating host physiology has emerged as an important area of research. Since oysters are the world’s most heavily cultivated bivalve molluscs, numerous studies have applied molecular techniques to understand the taxonomic and functional diversity of their associated bacteria. Here, we expand on this research by assessing the composition and putative functional profiles of prokaryotic communities from different organs/compartments of the black-lipped pearl oyster *Pinctada margaritifera*, a commercially important shellfish valued for cultured pearl production in the Pacific region. Seven tissues, in addition to mucous secretions, were targeted from *P. margaritifera* individuals: the gill, gonad, byssus gland, haemolymph, mantle, adductor muscle, mucus, and gut. Richness of bacterial Operational Taxonomic Units (OTUs) and phylogenetic diversity differed between host tissues, with mucous layers displaying the highest richness and diversity. This multi-tissues approach permitted the identification of consistent microbial members, together constituting the core microbiome of *P. margaritifera*, including *Alpha*- and *Gammaproteobacteria*, *Flavobacteriia*, and *Spirochaetes*. We also found a high representation of *Endozoicimonaceae* symbionts, indicating that they may be of particular importance to oyster health, survival and homeostasis, as in many other coral reef animals. Our study demonstrates that the microbial communities and their associated predicted functional profiles are tissue specific. Inferred physiological functions were supported by current physiological data available for the associated bacterial taxa specific to each tissue. This work provides the first baseline of microbial community composition in *P. margaritifera*, providing a solid foundation for future research into this commercially important species and emphasises the important effects of tissue differentiation in structuring the oyster microbiome.

## Introduction

Microbial symbionts play key roles in the survival, homeostasis and development of eukaryotic organisms (McFall-Ngai et al., 2013; Bang et al., 2018). Symbiotic associations between bacteria (among other microbes) and macroorganisms are ubiquitous in nature and have been the focus of extensive research. For instance, bacterial communities have been found to have profound impact on host nutrition, pathogen resistance and development of immune function, as well as other beneficial processes (Taylor et al., 2007; Kamada et al., 2013; Mueller and Sachs, 2015; Vandenkoornhuyse et al., 2015; Foster et al., 2017), while the eukaryotic host provides a stable environment and a constant nutrient supply for their associated bacteria. Previous studies have demonstrated that host-associated microbial community compositions are not stochastic, but mostly determined by host phylogeny and habitat (Cardenas et al., 2014; Pantos et al., 2015; Brooks et al., 2016; Carrier and Reitzel, 2018), and can be unevenly distributed within an individual host (Hernández-Agreda et al., 2016). Understanding the mechanics and importance of host-microbiome systems thus requires prior knowledge of both the distribution of microbial communities at the scale of the individual, and some assessment of the influence that microbiome composition may have on host functioning.

Rapid advances in sequencing technology and a decline in associated costs have spurred an increase in microbiome research, targeting a multitude of organisms and environments (Caporaso et al., 2010). Of these, the microbiota of marine invertebrates, including crustaceans, polychaetes, echinoderms, tunicates, sponges and corals, have been investigated extensively over the last two decades. These studies have demonstrated that many marine invertebrates host diverse, core microbial communities that are phylogenetically distinct from those of surrounding waters (Moitinho-Silva et al., 2014; Lokmer et al., 2016b; Zhang et al., 2016; Lu et al., 2017), and that these microbial associations can be host-specific regardless of geography (Reveillaud et al., 2014; van de Water et al., 2016; Brener-Raffalli et al., 2018). Nonetheless, there are several biological factors that can contribute to specific variation in microbial communities, such as host genetics and physiology (Jaenike, 2012; Wegner et al., 2013; Kohl and Carey, 2016; Amato et al., 2018), host health (Cárdenas et al., 2012; Lu et al., 2013; Sweet and Bulling, 2017), diet (Carrier et al., 2018), life stages (Trabal et al., 2012; Lema et al., 2014), and host–tissue differentiation (Meisterhans et al., 2016; Engelen et al., 2018; Høj et al., 2018).

Studies on microbiota of bivalves are of particular interest because of their importance in ecosystem functioning, but also due to their economic value and consumption by humans. Oysters are one of the most widely consumed and most valuable of all bivalve molluscs exploited commercially (Dumbauld et al., 2009), making them a relevant model for investigating host–microbiome systems. As such, microbial associations in oysters have been well documented, though the vast majority of the literature focuses on the role of associated microbial communities in preventing colonisation and establishment of pathogens (Lokmer and Wegner, 2015; Green et al., 2018; King et al., 2018; Laroche et al., 2018). Bacterial communities can vary as a function of tissue types in many organisms (e.g., bivalves, sponges, corals, plants, and humans) due, among others, to differences in host cell functions and/or composition of cellular components (Costello et al., 2009; Antunes et al., 2010; Schmitt et al., 2012; Ainsworth et al., 2015; Coleman-Derr et al., 2016). As a consequence, tissue differentiation is thought to strongly influence the microbiome of oysters. Yet, most microbial surveys of oyster species have been performed on whole body homogenates (Beleneva et al., 2007) or single tissues (Trabal et al., 2012; Trabal-Fernández et al., 2014; Banker and Vermeij, 2018). Understanding oyster-microbial associations requires an understanding of organ compartmentalisation, since different oyster compartments (hereafter named tissues) may harbour unique microbial assemblages that are linked to the physiological role of each host compartment. To date, variations in the composition of microbial assemblages between oyster tissues has rarely been considered (but see King et al., 2012; Lokmer et al., 2016b). To fill this gap, we investigated and compared the prokaryote communities of different tissues in the black-lipped pearl oyster *Pinctada margaritifera*. This shellfish species is highly valued for cultured pearl production and its industry represents the second most valuable economic resource in French Polynesia after tourism (Ky et al., 2019). This oyster species can produce valuable black pearls by biomineralisation, following the insertion of a mantle graft from a donor into the gonad of a recipient oyster, together with a nucleus (Gervis and Sims, 1992; Taylor and Strack, 2008). While recent studies have investigated the genome, transcriptome (Le Luyer et al., 2019) and phenome (Ky et al., 2018) to identify potential links to cultured pearl quality traits in *P. margaritifera*, no data relating to its microbiome exists at present. Characterising the microbial composition of symbiont assemblages in *P. margaritifera* is critical, since these microbial communities likely play a role in maintaining oyster fitness (e.g., Lokmer et al., 2016b) and by extension, may subsequently impact upon pearl quality.

The aim of this study, therefore, is to provide a microbial baseline for *P. margaritifera* and determine the balance between a stable core microbiome and transient microbial components between pearl oyster tissue types. To do so, we characterised the composition, diversity and predicted functional roles of bacterial communities of eight tissues and secretions, including the gill, gonad, byssus gland, haemolymph, mantle, adductor muscle, mucus, and gut. Our study shows that bacterial community composition and underlying functional profiles can be readily distinguished across *P. margaritifera* tissues. Although microbial communities of the gill, gut and haemolymph have been well studied in oysters (among other bivalves), to our knowledge the present study provides the first description of the microbiota associated with oyster mucus, muscle and byssus gland. Our multi-tissues approach also led to the identification of consistent microbial members, including several tissue-specific bacterial taxa. Unexpectedly, it was found that many tissue microbiomes (e.g., gills, gonad and mantle, among others) were dominated by the symbiont *Endozoicomonas*, a bacterial genus that is also prominent in many coral reef animals, although nearly absent in other oyster species.

## Materials and Methods

### Sampling of *Pinctada margaritifera* Tissues

Wild *Pinctada margaritifera* individuals (*N* = 12) were collected in January–February 2016 in the lagoon of Takapoto atoll (14°32′ S, 145°14′ W, Tuamotu Archipelago, French Polynesia) during the natural spawning season. After 20 months of subsurface rearing (3–5 m below the surface) the pearl oysters (shell dorso-ventral measurements of 8.4 ± 1.8 cm) were removed from the spat collectors, pierced and tied together onto chaplets rearing systems, where they remained until their transfer to Ifremer facilities (Vairao, Tahiti, French Polynesia) in May 2018. Individuals were kept for one month in the lagoon at Vairao prior to the sampling of eight selected tissues and secretions: the gill, the gonad, the byssus gland, the haemolymph, the mantle, the adductor muscle, the mucus, and the gut (Supplementary Figure S1). To avoid differences in microbial community compositions as a function of oyster size, 12 individuals of similar sizes were selected to investigate the composition and functional characterisation of *P. margaritifera* microbiome. Pearl oysters were scrubbed and rinsed to remove any remaining debris on the outer shell and then shucked using shucking knives. For each oyster, 500 μL of haemolymph was collected in the byssus using a sterile syringe and the mucus via a sterile swap and preserved in absolute ethanol. These two aqueous tissues were collected prior to entirely opening the shells of each sampled animal. By doing so, we aimed to collect the mucous layer before inducing acute physiological stress, that typically results in the secretion of mucus in large amounts. Then, we opened the shells and dissected the animal to collect the remaining six tissues using sterile scalpel blades. Prior to transfer the samples in absolute ethanol, each sample was rinsed with 70% ethanol to reduce the possibility of cross-contamination between tissues from the large amounts of mucus secreted during dissection.

### DNA Extraction, Library Preparation and 16S rRNA Gene Sequencing

Prior to DNA extraction, all samples, with the exception of the haemolymph and mucus, were crushed using mortar and pestle until the tissue was completely homogenised and poured back in 15 ml falcons. Before processing the samples for homogenisation, each sample was once again rinsed with 70% ethanol to reduce the amount of mucus still present in the absolute ethanol that served to preserve the tissue. Each homogenised sample was centrifuged at 1,500 *g* for at least 45 s to separate the particulate matter from the ethanol and kept on ice until DNA extraction. For the mucus samples, swaps were soaked directly in PowerBeads tubes (included in PowerSoil DNA Kit, see below) for 10 min and gently vortexed before being removed. Remnant mucus and ethanol were centrifuged at 1,500 *g* for 10 min. Haemolymph samples were directly centrifuged at 1,500 *g* for 10 min. Pellets from centrifuged mucus and haemolymph samples were kept on ice until DNA extraction. DNA was extracted from homogenised tissues using a modified protocol of the Qiagen DNeasy PowerSoil Kit. About 0.5 g of homogenised tissues were added into the PowerBead tubes, while the entire sample was used for the gonad, mucus and haemolymph. A succession of three thermal shocks was performed prior to DNA extraction to facilitate the breakage of the bacterial outer membrane, each including three steps: (1) −80°C for 10 min, (2) 65°C for 5 min, and (3) 2 min of vortexing at maximum speed. Once all thermal shocks were completed, each sample were kept at room temperature for 10 min. Next, DNA extraction was processed according to manufacturer’s intructions with a 10 min heating step at 70°C as recommended in the troubleshouting guide. Extracted DNA was quantified using a Qubit dsDNA HS Kit (Invitrogen, Carlsbad, CA, United States). DNA was then shipped to the Génome Québec Innovation Centre (McGill University, Canada) for the preparation and sequencing of the 16S rRNA amplicon library. The V4 region of the 16S rRNA gene was amplified using Polymerase Chain Reactions (PCRs) of the primers 515F and 806R (Caporaso et al., 2010) with added sequencing adapters CS1 and CS2 (Ison et al., 2016) (forward: 5′–ACACTGACGACATGGTTCTACAGTGCCAGCMGCCGCGG TAA–3′; reverse: 5′–TACGGTAGCAGAGACTTGGTCTGGACT ACHVGGGTWTCTAAT–3′; CS1 and CS2 adaptor sequences are underlined). The library was prepared following three successive thermocycler runs for tagging the 16S rRNA gene and another run for barcoding the fragments. Fragments were then pooled and purified using AMPure XP beads (Beckman Coulter, Inc.) (Bronner et al., 2014), and library quality control was performed. The library was run on a MiSeq sequencing system using paired end read of 250 base pairs (PE250).

### Bioinformatic Analysis

Raw sequences were processed with mothur v.1.39.5 (Schloss et al., 2009). After paired end reads were assembled into contigs, sequences longer than 298 bp (97.5% cutoff) were removed for further analyses, as well as sequences containing ambiguously called bases. We filtered the dataset for singletons and aligned the remaining sequences for the V4 region of the SILVA reference database, allowing for up to a 2 nt difference between the sequences. Chimera reads were removed using the UCHIME program (Edgar et al., 2011) as implemented in mothur. Sequences were then classified taxonomically according to the Greengenes database^1^ with a minimum bootstrap value of 60%. Any sequences belonging to mitochondria, chloroplasts, and eukaryotes were removed. Bacterial sequences were subsequently subsampled and rarefied based on the sample having the smallest number of sequences, i.e., to 2,687 (as described in the MiSeq SOP protocol, Kozich et al., 2013). Subsampled sequences were clustered into Operational Taxonomic Units (OTUs) using the average neighbour algorithm method with minimum 97% of similarity (cutoff = 0.03) and 16S rRNA reference amplicon sequences were determined for each OTU (Supplementary Data S1).

### Statistical Analysis

Phylogenetically annotated 16S sequences were used to characterise bacterial community composition of each tissue type at the family level. Stacked-column barplots were generated using the package ‘ggplot2’ in R based on total abundances of OTU counts for mean abundances of a given bacterial family within each tissue type. Then, relative abundances of the 22 bacterial families, including the “Other” category, were calculated from these mean abundances. Potential differences in community composition as a function of tissue type were assessed at both the family and OTU level by a permutational multivariate analysis of variance (PERMANOVA) and analysis of similarities (ANOSIM) with the adonis and anosim functions in the package ‘vegan’ integrated in R. For those tissues and secretions that showed significant dissimilarity in bacterial community composition (pairwise adonis function in R, *P*-values with Bonferroni corrections), similarity percentage (SIMPER) analyses were then conducted in the R package ‘vegan’ to determine which families contributed most to the degree of dissimilarity between host tissues. Community diversity and richness, as described respectively by the Shannon diversity index and Chao-1 richness, were inferred at the OTU level using mothur. Differences in alpha diversity between tissues were assessed by an analysis of variance (ANOVA) for the Shannon diversity index and a Kruskal–Wallis test for Chao richness (since normality of residuals and homogeneity of variances were rejected, i.e., Shapiro and Bartlett tests, respectively). Similarity in the assemblage of bacterial OTUs (based on the 4,085 OTUs rather than their family assignments) between host tissues was also assessed by PERMANOVA and ANOSIM tests and results were visualised using a non-metric multidimensional scaling (nMDS) ordination plot. All statistical analysis were performed on Bray Curtis distances of log (x + 1) transformed OTU counts using R version 3.4.4. The core microbiome of *P. margaritifera* was identified by plotting the abundance of OTUs by the percentage of sample representation at 2% intervals (from 0 to 100%, as in Ainsworth et al., 2015). Based on this distribution frequency across samples (see Supplementary Figure S2), OTUs present in at least 70% of samples were chosen as members of the core microbiome and their sequences were BLASTed against GenBank database [nr/nt and 16S rRNA sequences (*Bacteria* and *Archaea*)] for closely related matches.

Next, we performed an indicator species analysis using the R package IndicSpecies (De Cáceres and Legendre, 2009) to identify bacterial taxa that significantly associate with distinct host tissues. The analysis was conducted on OTU counts with host tissues as the grouping variable. Only OTUs that displayed highly significant (*P* < 0.01) associations with one tissue type were considered as indicator OTUs. The heatmap was compiled based on abundances of OTU counts that were previously identified as being tissue specific using the IndicSpecies analysis. Graphical representation of these tissue-specific OTUs was compiled in R using ‘ggplot2’, as well as the stacked-column plots representing relative abundances of tissue-specific bacterial taxa grouped at the family level.

Finally, to predict the functionality of the metagenomic content of each tissue type, we applied a computational approach using the Phylogenetic Investigation of Communities by Reconstruction of Unobserved States software (PICRUSt version 1.0) (Langille et al., 2013) as implemented in the Galaxy web application^2^. Briefly, the input file of the OTU table was created in mothur using the command ‘make.biom’. The command ‘normalise_by_copy_number.py’ was applied to account for differences in genomic copy number and metagenome predictions were conducted using ‘predict_metagenomes.py’. Individual KEGG Ortholog groups (KOs) were summarised at KEGG pathway level 1, 2, and 3 with the command ‘categorise_by_function.py’. This table was in turn used as input for LEfSe (Segata et al., 2011). Using LEfSe method, we tested data for statistical significance, biological consistency, and effect size relevance of predicted KEGG pathways of bacterial communties between tissue types, based on an LDA threshold of 2.5 for levels 1–3 of individual KOs and significant cutoff < 0.05. Graphical representations of LEfSe results were generated using ‘ggplot2’ R package and are presented hierarchically using barplots for individual KOs that displayed highly significant (LDA > 2.5) associations with one tissue type. A weighted Nearest Sequenced Taxon Index (NSTI) score was also calculated for each sample to confirm the accuracy of this computational approach, which mostly depends on the availability of reasonably related reference genomes (Langille et al., 2013).

## Results

### Composition of *Pinctada margaritifera* Microbiome

Bacterial DNA was isolated from eight tissues and secretions (gill, gonad, byssus gland, haemolymph, mantle, muscle, mucus, and gut) collected from twelve individuals of *P. margaritifera*, totalling 95 samples (the muscle was not analysed for one individual). 16S rRNA gene sequencing yielded to 4,331,391 sequences that were further clustered into 4,085 distinct OTUs (Supplementary Data S1). *P. margaritifera* hosted 35 unique bacterial phyla, 103 classes, and 134 orders. The most abundant phylum recovered across all samples was the *Proteobacteria* (56.5%), followed by the *Bacteroidetes* (11.4%), *Firmicutes* (5.4%) and *Planctomycetes* (4.6%), and many other unclassified bacteria (13.4%). BLAST searches for representative sequences showed that the most abundant unassigned OTU (OTU0002, 10.2% of relative abundance across all samples) is related to *Spirochaetes* previously detected in marine invertebrates (Table 1).

**TABLE 1:** Summary of bacterial OTUs corresponding to the core microbiome of the pearl oyster *Pinctada margaritifera*.

**OTU**	**Number of reads**	**Relative abundance (%)**	**Lowest taxonomic level from Greengenes classification**	**Source nearest relative (GenBank accession number, Sequence identity; Host or environment)**
**Otu0001**	35 957	14.09	*Endozoicimonaceae*	Uncultured *Endozoicomonas* sp*.* (MG525087; 98%; Polychaete *Hydroides elegans*)
Otu0002	26 030	10.20	Bacteria unclassified	Uncultured *Spirochaetes* (KP174129; 89%; Anemone *Parazoanthus lucifium*)
Otu0005	6 095	2.39	Oceanospirillales unclassified	*Teredinibacter turnerae* (o_*Cellvibrionales;* f_*Cellvibrionaceae*) (KY643669; 93%; Shipworm *Kuphus polythalamia*)
Otu0006	5 832	2.28	Aeromonadales unclassified	Uncultured *Vibrio* sp. (o_*Vibrionales*; f_*Vibrionaceae*)** (MG554521; 100%; Bivalve *Gari maculosa*)
**Otu0008**	4 753	1.86	*Rhodobacteraceae* (genus *Roseivivax*)	Uncultured *Ruegeria* sp.** (MG525075; 100%; Polychaete *Hydroides elegans*)
Otu0014	2 891	1.13	*Flavobacteriaceae* (genus *Tenacibaculum*)	*Tenacibaculum* sp. (KY655362; 100%; Sponge *Pione vastifica*)
Otu0016	2 481	0.97	*Pseudoalteromonadaceae* (*Pseudoalteromonas porphyrae*)	*Pseudoalteromonas* sp.** (GQ391981; 100%, Diseased gorgonian *Pseudoalteromonas americana*)
Otu0026	1 233	0.48	*Colwelliaceae*	*Colwelliaceae* bacterium (FJ952829; 100%; Coral *Montastrea annularis*)
Otu0027	1 195	0.47	*Alteromonadaceae* (genus *Glaciecola*)	Uncultured *Alteromonadaceae* (KC917980; 100%; Crab *Callinectes sapidus*)
Otu0030	1 107	0.43	*Alteromonadaceae* (genus *Microbulbifer*)	*Microbulbifer* sp. (KC854344; 100%; Sponge *Theonella swinhoei*)
Otu0046	891	0.35	*Rhodobacteraceae* (genus *Dinoroseobacter*)	*Pseudooceanicola lipolyticus* (f_Rhodobacteraceae) (KY273603; 99%; Sea water Western Pacific Ocean)

*In bold are the OTUs that were detected in more than 90% of all pearl oyster samples.*

Although no OTU was found across all samples (100% sampling coverage), eleven conserved bacterial OTUs (present in at least 70% of all samples) were identified as ubiquitous members of the putative core microbiome, regardless of their relative abundances and tissue association (Table 1). *Gammaproteobacteria* was the most dominant taxa, representing 63.6% of the core microbiome. Two highly conserved core members were recovered across 90% of all pearl oyster samples, one was of the *Endozoicimonaceae* family and another of the genus *Roseiviax* (OTUs 0001 and 0008 respectively, Table 1). The majority of bacterial taxa identified as members of the core microbiome were present at low abundances (<5%).

### Bacterial Community Composition Differs Between Pearl Oyster Tissues

**FIGURE 1:**
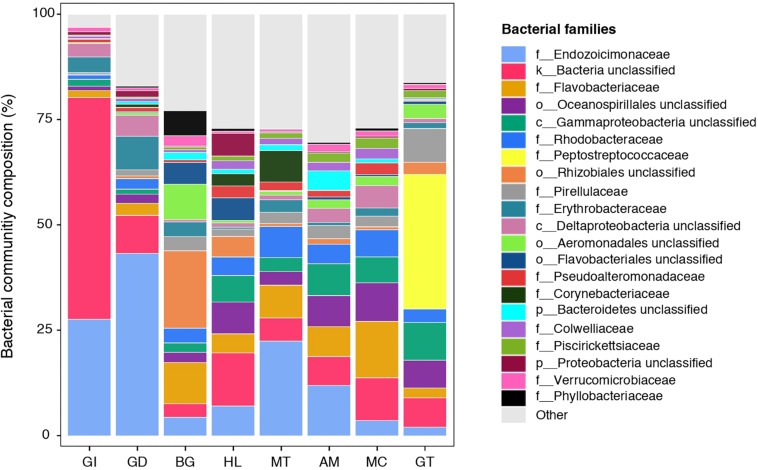
Bacterial community composition of the pearl oyster *Pinctada margaritifera*. Average relative abundances of the most represented families are shown for each of the eight tissue types; gill (GI), gonad (GD), byssus gland (BG), haemolymph (HL), mantle (MT), adductor muscle (AM), mucus (MC), and gut (GT). Each colour represents a distinct family level OTU or lowest level available. The 21 dominant families are indicated. Less commun families were grouped as ‘Other’ and are shown in light grey.

**FIGURE 2:**
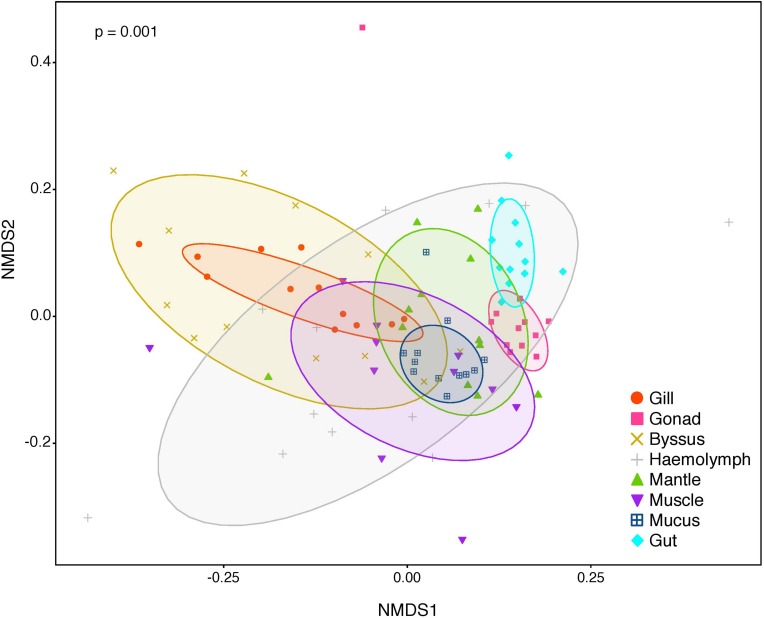
Non-metric multidimensional scaling (nMDS) of bacterial community composition of *P. margaritifera* at the OTU-level. Each symbol colour denotes one tissue (Gill = red, Gonad = pink, Byssus gland = gold, Haemolymph = grey, Mantle = green, Muscle = purple, Mucus = blue, Gut = cyan), ellipses are drawn around each group’s centroid (95%). *P*-values indicate significant differences in bacterial community composition between tissues (PERMANOVA and ANOSIM tests).

**FIGURE 3:**
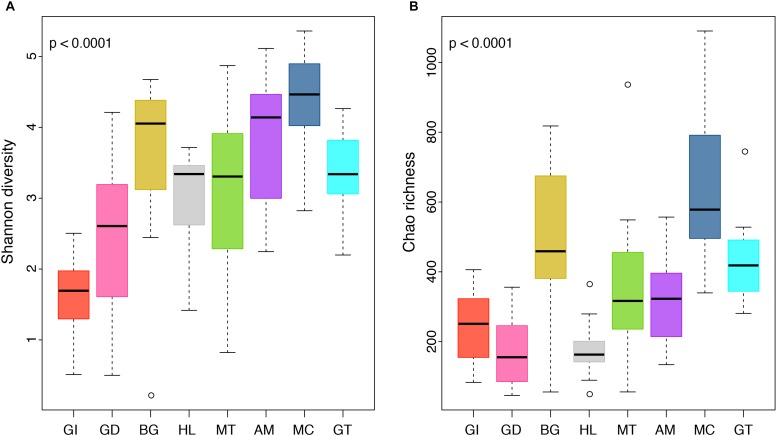
Boxplot displaying differences in community diversity between *P. margaritifera* tissues. Shannon diversity **(A)** and Chao richness **(B)** values are shown for each of the eight tissue types; gill (GI), gonad (GD), byssus gland (BG), haemolymph (HL), mantle (MT), adductor muscle (AM), mucus (MC), and gut (GT). Boxes display the first and third quartile spread of the data, with the line in the box indicating the median, the whiskers denoting the minimum and maximum values and open dots as outliners of the data. *P*-values indicate significant differences in community diversity between tissues (ANOVA and Kruskal–Wallis tests).

The composition of tissue-specific microbiomes in *P. margaritifera* displayed significant differences between tissue types, based on relative abundances of bacterial families (Figure 1; PERMANOVA: pseudo-*F* = 4.6471, *p* = 0.0001; ANOSIM: *R* = 0.3646, *p* = 0.0001), individual OTUs (Figure 2; PERMANOVA: pseudo-*F* = 3.6285, *p* = 0.0001; ANOSIM: *R* = 0.4407, *p* = 0.0001) and community diversity and richness (Figure 3; ANOVA and Kruskal–Wallis: *p* < 0.0001, respectively). However, only OTU compositions in the gill, byssus gland and gut differed significantly from all other tissues based on the number of individual OTUs reads (PERMANOVA: *p* < 0.05; Supplementary Data S2).

**FIGURE 4:**
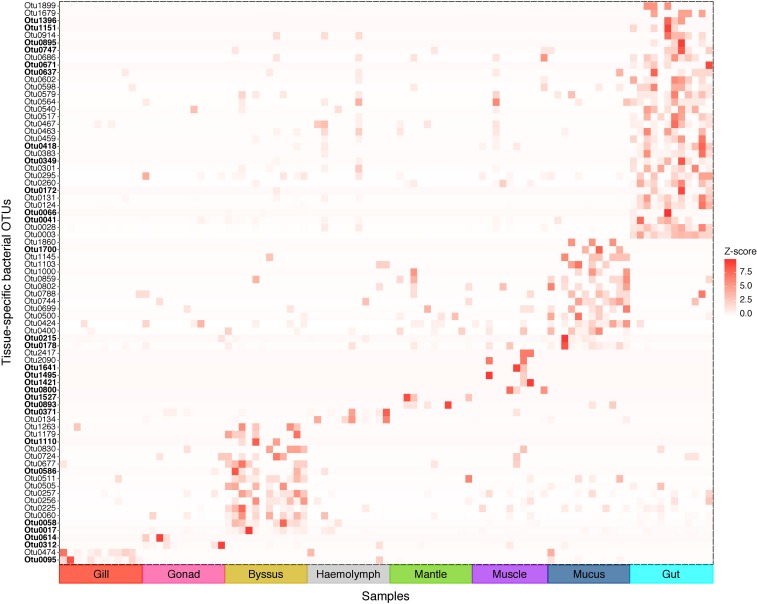
Indicator bacterial OTUs for variation in bacterial communities between *P. margaritifera* tissues. Heatmap based on IndicSpecies analysis shows specific bacterial OTUs that characterise each tissue. Each cell represents the standard transformation of the counts for each specific OTU per tissue. Bold OTUs indicate highly tissue-specific OTUs with *Z*-scores > 7.5. See Supplementary Data S4 for details on IndicSpecies analysis and *Z*-score values.

**FIGURE 5:**
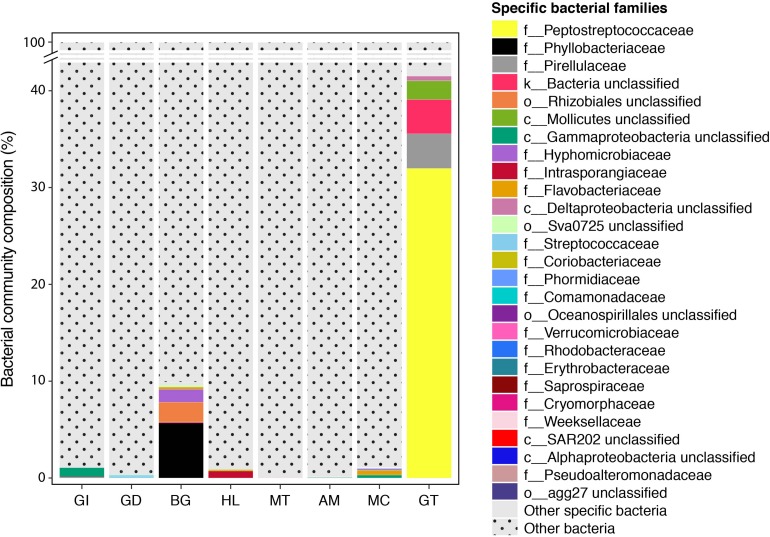
Indicator bacterial families for variation in bacterial communities between *P. margaritifera* tissues. Specific bacterial families associated with each tissue. Bar plots represent the average relative abundance of each identified specific bacterial OTU at the family level; dotted bars are none specific OTUs.

We further analysed our data for the presence of tissue-specific bacterial OTUs that characterised microbial variations between *P. margaritifera* tissues. Each tissue was consistently associated with a set of tissue-specific bacterial OTUs (Figure 4), most of which were detected in low abundances (<1%; Figure 5). The number of tissue-specific OTUs ranged from 2 to 32 (mean: 9.5 and median: 4.0) and their phylogenetic membership varied between tissues (Figure 5 and Supplementary Data S4). Tissue-specific bacterial OTUs represented less than 2% of the microbiomes associated with the gill, gonad, haemolymph, mantle, muscle, and mucus, while they represented nearly 10% of the byssus gland bacterial community and over 40% for the gut (Figure 5).

The gill microbiome displayed a higher relative abundance of unclassified bacteria (52.6%; Figure 1) (SIMPER: *p* < 0.05; Supplementary Data S3), of which nearly all reads (99.0%) were identified as *Spirochaetes* based on BLAST searches. OTUs classified as belonging to *Endozoicimonaceae* were notably abundant in the gill (27.6%; Figure 1). This bacterial family was detected in all oyster tissues, although at lower relative abundances, apart from the gonad in which *Endozoicimonaceae* dominated the microbial community (43.3%; Figure 1). Two tissue-specific OTUs of unclassified *Gammaproteobacteria* and bacteria (Figures 4, 5) were identified in the gill corresponding to *Endozoicomonas* (NCBI accession number MG525087; 92% sequence identity) and *Arcobacter* (KF721482; 79% sequence identity) based on BLAST searches.

In the gonad, the bacterial dominance by OTUs belonging to *Endozoicimonaceae* resulted in low values of evenness (Shannon) and species richness (Chao) (Figure 3), as well as in a tight cluster in the nMDS plot (Figure 2). The gonad also had higher relative abundance of *Erythrobacteraceae*-related sequences (7.9%; Figure 1), as well as many other rarer families (<1%), including the *Streptococcaceae*, *Moraxellaceae*, *Bacillaceae*, and *Brevibacteriaceae* (Supplementary Data S3). The gonad microbiome displayed two tissue-specific OTUs classified as *Streptococcus* and *Alicycliphilus* (*Comamonadaceae*) (Figures 4, 5 and Supplementary Data S4).

The byssus gland had a microbiome with relatively high alpha diversity (Figure 3). This was reflected in high proportions of bacterial families with relative abundances < 1% (25.6%; Figure 1). Many of these rare bacterial families were found in higher abundances within this tissue, including *Hyphomicrobiaceae*, *Sphingomonadaceae*, *Microthrixaceae* (Supplementary Data S3), as well as abundant OTUs belonging to *Phyllobacteriaceae* (5.9%) and unclassified *Rhizobiales* (18.3%; Figure 1). About seven percent of the byssus gland microbiome were represented by one tissue-specific OTU that belonged to the family *Phyllobacteriaceae*, while the remaining fourteen were mostly of unclassified *Rhizobiales* and *Hyphomicrobiaceae* (Figure 5).

The haemolymph microbiome displayed a low value of species richness (Figure 3), in addition to increased abundances of many rare bacterial families, such as *Intrasporangiaceae* and *Coriobacteriaceae* (including two tissue-specific OTUs belonging to these bacterial families; Figures 4, 5), as well as *Dermabacteraceae* and *Prevotellaceae* (Supplementary Data S3, S4).

The gut microbiome was dominated by a single OTU classified as *Peptostreptococcaceae* (32.0%; Figure 1), resulting in a tight cluster in the nMDS plot (Figure 2). Despite this bacterial dominance, the gut had relatively high values of alpha diversity (Figure 3). This was reflected in the high proportions of *Pirellulaceae* (8.0%; Figure 1) and many other rare families (e.g., *Synechococcaceae*, *Mycobacteriaceae*, *Desulfobulbaceae*, and unclassified *Mollicutes*) (Supplementary Data S3). Thirty-one percent of the gut microbiome were represented by a single tissue-specific OTU related to *Peptostreptococcaceae*, about 8% by unclassified bacteria and *Pirellulaceae* (5 and 8 OTUs, respectively), and 2% by two OTU related to unclassified *Mollicutes* (Figure 5 and Supplementary Data S4).

Microbiomes associated to the mucus, mantle and muscle were highly similar based on relative abundances of bacterial families and individual OTUs (PERMANOVA: *p* > 0.05), with high proportions of bacterial families with relative abundances < 1% (30.3, 29.9, and 32.7% for the mucus, mantle, and muscle respectively; Figure 1). Of these rare families, *Oceanospirillaceae*, *Hyphomonadaceae* and *Haliangiaceae*, among others, had significant higher proportions in the mucus compared to all other tissue types, while *Syntrophobacteraceae*, *Kordiimonadaceae*, and *Spirochaetaceae* had significant higher proportions within the muscle (Supplementary Data S3). The mucus microbiome also had the highest values for both evenness and species richness (Figure 3), as well as the highest relative abundance of OTUs belonging to *Flavobacteriaceae* (13.4%; Figure 1). The mucous layer had a tissue-specific microbiome of fifteen OTUs dominated by the *Flaovobacteriaceae* (Figure 5), including the OTU belonging to unclassified *Gammaproteobacteria* with the closest sequence matching *Ascidianibacterium* (AB377123; 98% of sequence identity). The mantle microbiome displayed relatively high abundances of *Endozoicimonaceae* and *Corynebacteriaceae* (22.5 and 7.8%, respectively; Figure 1), and only four rare families had higher proportions within this tissue (HTCC2089, Ellin6075, *Oscillatoriophycideae* and unclassified BPC015) (Supplementary Data S3). Two tissue-specific OTUs belonging to the bacterial families *Verrucomicrobiaceae* and *Saprospiraceae* were also identified in the mantle (Figures 4, 5), while the muscle microbiome had six tissue-specific OTUs, among which the most abundant was an unclassified *Gammaproteobacteria* identified as *Oceanospirillaceae* (EU167355; 96% sequence identity) based on BLAST searches.

### Tissue-Specific Bacterial Communities Display Distinct Functional Profiles

**FIGURE 6:**
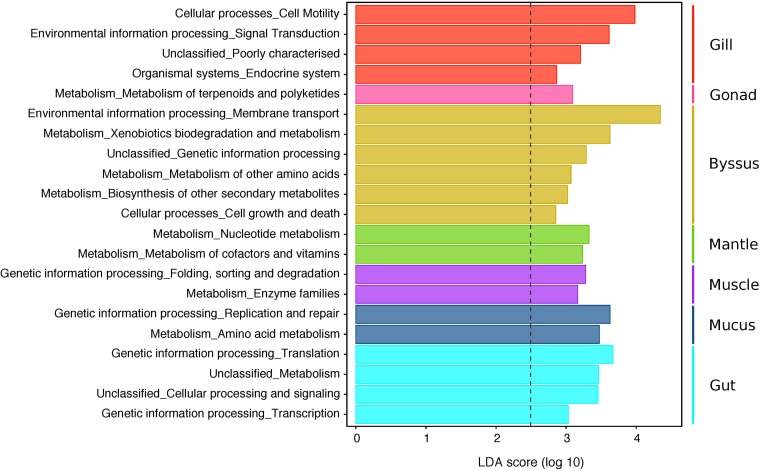
LEfSe analysis displaying predicted functional profiles of the different tissue types in *P. margaritifera*. The barplot shows the 21 differentially abundant KEGG pathways (level 2) identified in the microbiomes of particular host tissues, based on a Linear Discriminant Analysis (LDA, *p* < 0.05 for factorial Kruskal–Wallis and pairwise Wilcoxon tests). Each colour denotes the tissue type, wherein the distinguishing functional traits were identified (Gill = red, Gonad = pink, Byssus gland = gold, Mantle = green, Muscle = purple, Mucus = blue, Gut = cyan). Only KEGG pathways meeting an LDA significant threshold > 2.5 are shown. The threshold of the logarithmic LDA score is represented by the dotted line. See Supplementary Data S6 for abundances of predicted gene counts of KEGG pathways (level 1 to 3).

Functional profiles of the 4,085 OTUs were determined using a predictive metagenomic analysis to identify putative functions and processes underlying distinct microbial communities associated with specific host tissues. Mean NSTI scores varied from 0.11 (SD ± 0.03) and 0.15 (SD ± 0.02) depending on the tissue types (Supplementary Data S5). These values were within the range of mammal microbiomes that have been previously predicted with reasonable accuracy, though well covered microbiome samples are generally below 0.05 (Langille et al., 2013). After metagenomic predictions were summarised at the level 2 of KOs, LEfSe analysis showed significant differences in predicted functional profiles with 21 distinguishing traits, identified in the microbiomes of particular host tissues (Figure 6 and Supplementary Data S6). Generally, PICRUSt and LEfSE analyses suggested significant differences in several predicted metabolic pathways of distinct bacterial community composition characterising the gonad, mantle and mucus. These differences were mostly through enrichment in genes encoding for pathways related to different metabolisms (e.g., terpenoids and polyketides, nucleotides, cofactors and vitamins, amino acids, and enzyme families). Metagenomic predictions also suggested that genes associated with the endocrine system, cell motility and signalling functions were the most abundant metabolic categories for the gill microbiome, while the byssus gland microbiome was enriched in genes affiliated to xenobiotic, amino acid and secondary-metabolite metabolisms, together with membrane transport and cell growth and death. The gut was mostly enriched in genes related to genetic information processing, including translation and transcription. No predicted functional trait was identified for the bacterial community associated with the haemolymph.

## Discussion

The present investigation is the first report that characterises the microbiome and tissue-specific bacterial composition of the black-lipped pearl oyster *Pinctada margaritifera*. Specifically, the objectives underpinning this study were two-fold: (1) providing the first description of consistent and dominant microbial members of *P. margaritifera* that can then be targeted in future function-based investigations of bacterial community composition for the potential improvement of cultured pearl quality, and (2) testing the hypothesis that different functional host compartments (i.e., different tissues and secretions) of *P. margaritifera* harbour different microbial communities and display distinct inferred functional profiles.

### The Microbiome of *Pinctada margaritifera*

Similar to the microbiome of many oysters (Najiah et al., 2008; Green and Barnes, 2010; Fernández-Piquer et al., 2012; Cleary et al., 2015; Asmani et al., 2016; Pierce et al., 2016; Banker and Vermeij, 2018; Laroche et al., 2018), *P. margaritifera* microbial communities were dominated by the largest and most phenotypically diverse bacterial phylum, the *Proteobacteria*. The prevalence of this phylum was mostly attributable to *Alpha*- and *Gammaproteobacteria*. *Alphaproteobacteria* were identified as members of the *P. margaritifera* core microbiome, in particular OTUs related to aerobic heterotrophs from the *Rhodobacteraceae* family (*Roseivivax*_OTU0008 and *Dinoroseobacter*_OTU0046). *Roseivivax* and *Dinoroseobacter* are bacterial genera that are known to metabolise organic sulphur compounds, including dimethylsulphoniopropionate (DMSP), as well as carbon monoxide, a pathway often coupled with sulphate respiration processes (Gonzalez et al., 1999, 2000; Ansede et al., 2001; Zubkov et al., 2001; Rabus et al., 2006). These bacteria are also thought to have a great diversity of physiological properties, ranging from the degradation of lignin, aerobic oxidation of sulphites and production of antibiotics to aerobic anoxygenic photosynthesis (Buchan et al., 2005; Dang et al., 2008; Tang et al., 2010). Recovered *Endozoicimonaceae* (*Gammaproteobacteria*) sequences were in high abundances in the black-lipped pearl oyster (from 2.0 to 43.3% depending on the tissue) and present in at least 90% of all samples irrespective of the tissue (OTU0001). These bacterial symbionts have not previously been reported from other oyster species, but are dominant members of the core microbiome of several marine invertebrates (Neave et al., 2016). *Endozoicomonas* have been suggested to have important functional roles in their hosts related to nutrient acquisition, including host-associated protein and carbohydrate transport and cycling, and the production of amino acids and antimicrobial compounds (Neave et al., 2017). Other *Gammaproteobacteria*-related OTUs were identified as core members, including *Alteromonadaceae* and *Pseudoalteromonadaceae*, although observed at lower abundances across all tissues (<1%). *Alteromonadaceae* (e.g., *Glaciecola* and *Microbulbifer* genera) have been reported to play a role in oyster larvae development (Laroche et al., 2018), while *Pseudoalteromonas porphyrae*, and more specifically its associated oxidoreducting enzymes, are involved in plant growth and stress tolerance (Dimitrieva et al., 2006). Further investigations are thus needed to determine whether these bacteria play similar roles in *P. margaritifera*.

A high representation of *Bacteroidetes* in *P. margaritifera*, and more specifically *Flavobacteriia*, is in accordance with previous studies on oyster-associated bacterial community composition (Hernández-Zárate and Olmos-Soto, 2006; Zurel et al., 2011; Fernández-Piquer et al., 2012; Fernández-Gómez et al., 2013). These marine bacteria have been implicated in nutrient uptake via the production and/or remineralisation of organic matter ingested or produced by their hosts (Agostini et al., 2012), but also in biofilm formation (Ríos-Castillo et al., 2018), algal polysaccharide degradation (Mann et al., 2013), and infectious diseases (Nematollahi et al., 2003). Of note is the identification of the genus *Tenacibaculum* (*Flavobacteriaceae*) as a member of *P. margaritifera* core and stable microbial community. *Tenacibaculum* species are known pathogens reported in diseased oysters, and to have caused considerable economic losses in mollusc aquaculture (Collado et al., 2014; Burioli et al., 2018). These bacteria do not seem to express pathogenic capacity for infecting the French Polynesian pearl oyster (at least for the twelve individuals collected in our study, as they appeared healthy and showed no sign of infection), suggesting that *Tenacibaculum* may switch from being a commensal species to an opportunistic pathogen only under particular conditions (such as the presence of parasites; see Burioli et al., 2018). Furthermore, *Tenacibaculum* sp. have been pinpointed as being indicative of the reef flat environment (van Oppen et al., 2018), the habitat in which *P. margaritifera* oysters are grown and kept for pearl production. Oyster samples also returned high relative abundances of one core bacterial OTU identified via BLAST searches as belonging to the phylum *Spirochaetes* (OTU0002). Members of this phylum can play an important role in host nutrition, as well as in the production of antimicrobials in some gorgonian corals (van de Water et al., 2016; Wessels et al., 2017), but can also be causative agents of Akoya oyster disease (*Pinctada fucata martensii;* Matsuyama et al., 2017).

### Tissue-Specific Microbiome and Functional Profiles

As in all suspension-feeding bivalves, *P. margaritifera* uses its gills to pump water into the pallial cavity to capture, process and transport food particles (Beninger et al., 1997; Beninger and Veniot, 1999). Microbial analysis and BLAST searches revealed that the gill microbiome was dominated by *Endozoicomonas*- and *Spirochaetes*-related OTUs (*Endozoicimonaceae*_OTU0001 and Unassigned_OTU0002), a bacterial genus and phylum known for their roles in host nutrient acquisition and antimicrobial production (van de Water et al., 2016; Neave et al., 2017; Wessels et al., 2017). Inferred functional profile of the gill was characterised by higher relative abundances in genes encoding for pathways related to the endocrine system, cell motility and signal transduction, and those encoding for protein kinases (Supplementary Data S6), a key cytoplasmic signal transducer in immune cell signalling (Lim et al., 2015). Furthermore, BLAST searches identified a gill-specific bacterial OTU belonging to *Arcobacter*, a genus that has been implicated in pathogen defence and acclimation (Defer et al., 2013; Lokmer and Wegner, 2015). Together, these findings suggest that *P. margaritifera* has the potential to select specific bacteria through filtration by the gills. This selection process may target bacteria that are able to produce enzymes and antimicrobials involved in oyster immunity, functions that have been previously documented for bacterial communities associated to the gills of other oyster species (Zurel et al., 2011; Roterman et al., 2015) and marine bivalves (Antunes et al., 2010; Meisterhans et al., 2016).

Similar to the gills, the mucus is involved in the transport of particles during feeding and cleansing processes. In marine invertebrates, the mucus also serves as a physical and biological barrier against pathogens (Espinosa et al., 2016), and play a role in locomotion (Davies and Beckwith, 1999), adhesion (Smith et al., 1999) and nutrition (Ward and Shumway, 2004). In the present study, the mucus of *P. margaritifera* was characterised by the highest bacterial richness and diversity, and was dominated by *Flavobacteriaceae* and *Oceanospirillales* (including six tissue-specific OTUs), both reported as major bacterial components in coral mucus (Taniguchi et al., 2015; Badhai et al., 2016). The mucus microbiome was predicted to be enriched in genes encoding for pathways related to amino acid metabolism and carbon fixation, together with DNA replication and repair. These predicted functions are in accordance with the results of a previous study on the composition and functional characterisation of microbiome associated with coral mucus (Badhai et al., 2016). Using a true functional analysis of the metagenome, the authors accurately deciphered the role of the mucus microbiota in host nutrient uptake and protection against physicochemical injuries and virulence. The mantle, muscle and mucus microbiomes were highly similar despite a reduced abundance of *Endozoicimonaceae* in the mucus. Mantle tissues also returned significantly higher abundances of OTUs belonging to the genus *Corynebacterium* compared to all other tissue types. The role of these bacteria in marine invertebrates is unknown, but they have been reported to use urea as a nitrogen source (Siewe et al., 1998) and to produce metabolites, especially antibiotics (Dickschat et al., 2010).

In marine invertebrates, the haemolymph is a critical site in host immune response. Similar to other oysters, *P. margaritifera* haemolymph was characterised by a low bacterial richness, albeit still phylogenetically diverse (Potgieter et al., 2015) comprising relatively high abundances of *Spirochaetes*, *Flavobacteriia*, *Alpha*- and *Gammaproteobacteria* (Lokmer and Wegner, 2015; Lokmer et al., 2016a). Although *Vibrio*, *Acinetobacter*, and *Aeromonas* pathogens have been identified as prevalent groups of the haemolymph in some aquatic invertebrates (Zhang et al., 2018), including oysters (Vezzuli et al., 2018), these bacteria occurred in low abundances in *P. margaritifera* microbiome. In the present study, we were not able to confirm that the presence of the core members *Pseudoalteromonas, Endozoicomonas* and *Rhodobacterales* (among others) were involved in the production of antimicrobials against these pathogenic bacteria (as reported by Boöhringer et al., 2017; Vezzuli et al., 2018; Zhang et al., 2018), but such discrepancies warrant further exploration. The persistence of relatively rare, presumably resident bacteria, such as *Intrasporangiaceae* and *Coriobacteriaceae*, indicates that these microorganisms can resist the host immune system and might provide benefits to their host functioning or be commensal symbionts.

Gonad tissues of non-grafted *P. margaritifera* (i.e., no biomineralisation for pearl production) were largely dominated by *Endozoicimonaceae*, suggesting that these bacteria also play a role in oyster reproduction. *Erythrobacteraceae*, among other *Proteobacteria*, were prominent members of the gonad microbiome. These bacteria are facultative photoheterotrophs, metabolising a variety of organic carbon compounds, including glucose, pyruvate, acetate, butyrate, and glutamate (Tonon et al., 2014). They are also thought to be involved in coral reproduction (Ceh et al., 2012; Liang et al., 2017) and may play a similar role in oysters. Other gonad-specific bacteria, such as *Streptococcaceae* and *Comamonadaceae*, are involved in nutrient metabolism, and specifically in sulphur cycling (Tian et al., 2014; Gauthier et al., 2016). Predicted analysis of metagenome revealed that the gonad microbiome was enriched in genes encoding for pathways related to carbon cycling through aerobic mineralisation of some aromatic compounds (e.g., benzoate and naphthalene) and metabolisms of terpenoids, polyketides, and beta-alanine (Supplementary Data S6). These findings suggest that the gonad microbiome can be taxonomically and functionally diverse, and provide a first survey on the distribution of various predicted metabolic pathways previously unexplored in this reproductive tissue. Future investigations of grafted gonads would be highly valuable to elucidate the effects of the grafting process on microbial community composition. For instance, some bacteria can be introduced into the gonad during the grafting operation and depreciate the commercial value of pearls by negatively affecting the biomineralisation process and pearl quality traits (e.g., the occurrence of depressed rings and superficial depressed spots) (Cuif et al., 2018).

In the alimentary tissue, our microbial analysis revealed that *P. margaritifera* gut was dominated by *Peptostreptococcaceae* (phylum *Firmicutes*), *Pirellulaceae* (*Planctomycetes*) and *Mollicutes* (*Tenericutes*). These bacterial phyla are commonly associated with animal guts, including oysters (Green and Barnes, 2010; King et al., 2012; Lokmer et al., 2016b). For instance, species of the genus *Tepidibacter* (OTU0003) have been implicated in the decomposition of organic matter produced by organisms colonising deepsea hydrothermal vents, while other members of *Peptostreptococcaceae* and related genera were found in human intestines (Slobodkin, 2015). *Pirellulaceae* species have also been reported to exploit sulphated algal polysaccharides (Glockner et al., 2003), compounds that are commonly ingested by oysters as a consequence of phytoplankton consumption. Although *Mollicutes* have been previously reported in oyster guts (King et al., 2012), as well as in other invertebrate and fish guts (Kellogg et al., 2009; Nechitaylo et al., 2009; Huang et al., 2010), relatively little is known about their role in digestive systems. In this study, we observed an enrichment of predicted genes encoding for pathways related to various metabolisms, including pyrimidine, methane and sphingolipid, as well as high abundances of those encoding for the production of amino acids and vitamins, degradation of polysaccharides, and other genetic information processing, such as transcription and translation (Supplementary Data S6). Such an inferred functional profile could align with the contribution of gut microbial symbionts to heterotrophic metabolism and nutrient uptake, though ground-truthed metagenomic and/or metatranscriptomic data are needed to confirm these predicted metabolic functions.

The byssus gland, a compartment involved in oyster adhesion through secretions that form the byssal threads, had microbiomes mostly dominated by *Rhizobiales*, including tissue-specific *Phyllobacteriaceae* and *Hyphomicrobiaceae* species. *Bacteroidetes*, and particularly *Flavobacteriaceae*, were also prominent microbial members of the byssus gland. These bacteria have been implicated in organic matter degradation, such as cellulose and pectin (Thomas et al., 2011). Consequently, *Bacteroidetes* are often found in the guts of animals, where the secretion of a range of extracellular enzymes help the degradation of the decomposed organic matter. Strikingly, however, *Flavobacteriia* were found in low abundance in *P. margaritifera* gut, although prevalent in the byssus gland and mucus. This result highlights the need for more detailed studies to elucidate the role of these marine bacteria in oysters. Nonetheless, the predicted enrichment in genes involved in various metabolic pathways, including amino acids, metabolites and xenobiotics, as well as genes encoding for membrane transport, cell growth and death, may suggest that bacterial communities of the byssus gland are not only taxonomically diverse, but also display a variety of functional roles.

## Conclusion

The present study provides a snapshot of the microbial community composition and predicted functions in eight distinct tissues and secretions of the pearl oyster *P. margaritifera*. Several bacteria of the phylum *Proteobacteria*, *Bacteroidetes*, and *Spirochaetes* were identified as members of the core microbiome of the black-lipped pearl oyster *P. margaritifera*, regardless of their tissue affiliation. Such a stability in microbial communities between tissues implies that there is no tissue-specific condition that favours the growth of these microbial species. Above all, it is important to mention that core OTUs identified in the current study may also be ubiquitous due to the presence of overlying mucous layer, acting as a vector for bacterial transfer between the mucus and the tissues sampled, and this despite our efforts to minimise cross-contamination during dissection. Among these core members, many have been previously identified as prevalent microbial symbionts of several oyster species, including bacterial families of *Rhodobacteraceae, Alteromonadaceae, Pseudoalteromonaceae*, and *Flavobacteriaceae* (Asmani et al., 2016; Banker and Vermeij, 2018; Laroche et al., 2018). Conversely, *Endozoicimonaceae* and *Spirochetes* were identified for the first time as core members of oysters, in addition to being further prevalent in *P. margaritifera* gills and gonads. Such a novelty may be linked to host geography and habitat rather than host specificity. In fact, these bacteria are often dominating microbiomes of marine invertebrates inhabiting coral reef environments (van de Water et al., 2016; Neave et al., 2017; Wessels et al., 2017). Given the changeable state of the microbiome, meta-microbiome changes with time, seasons, locations and host age would be required to validate those core members identified in the current study (as described in Hernández-Agreda et al., 2017). One striking result is the identification of *Streptococcaceae* and *Comamonadaceae* as gonad-specific bacterial taxa, where both are known to be involved in sulphur cycling. Given that some bacteria are contributing to the formation of biochemical blends that can further impact the biomineralisation process of grafted pearl oysters (Cuif et al., 2018), function-based studies will be of great interest to investigate whether and how these sulphuric bacteria may depreciate pearl quality. Futhermore, our study highlights that, in addition to a ubiquitous core microbiome, bacterial community compositions vary between some tissue types, with certain bacterial taxa that seem to support their respective physiological functions. Consequently, the eight tissues surveyed in this study represent bacterial niches, with the gill, byssus gland and gut having the most distinct microbial assemblages, thus revealing a tissue-specific microbial consortium as reported in humans (Turnbaugh et al., 2007; Costello et al., 2009; Faust et al., 2015) and several marine invertebrates (King et al., 2012; Lokmer et al., 2016b; Høj et al., 2018). Overall, this study provides new perspectives on the largely understudied black-lipped pearl oyster-associated microbiome and directions for future studies.

## Data Availability

Raw sequencing data determined in this study are available under the NCBI BioProject ID PRJNA544968 (https://www.ncbi.nlm.nih.gov/bioproject/544968) for 16S rRNA gene data. Other data are available in the Supplementary Information.

## Ethics Statement

The collection of *P. margaritifera* followed institutional and national guidelines. The study was approved by the ethics committee of Ifremer.

## Author Contributions

CD, C-LK, and SP designed the research. CD performed the sampling, laboratory work, data analysis, and wrote the manuscript. C-LK and SP funded the research and reviewed draughts of the manuscript.

## Conflict of Interest Statement

The authors declare that the research was conducted in the absence of any commercial or financial relationships that could be construed as a potential conflict of interest.

## Footnotes

1 http://greengenes.secondgenome.com/downloads/greengenes_database/gg_13_5

2 http://huttenhower.sph.harvard.edu/galaxy/

## Supplementary Material

FIGURE S1 *P. margaritifera* compartments/tissues. Photo of each of the two shell valves showing, on the right, the collected tissues: GI, the gills; GD, the gonad; BG, the byssus gland; MT, the mantle; AM, the adductor muscle; and GT, the gut (shown partially on the photo). The haemolymph was collected from the byssus using a sterile syringe, while the mucus was collected on the mantle using a sterile swap.

FIGURE S2 Plotted bacterial OTU abundance in core microbiome’s of 0 to 100% of samples of the pearl oyster *P. margaritifera*.

DATA S1 Overview over sequence counts, taxonomic classification, and 16S rRNA reference amplicon sequence for all 4,085 OTUs.

DATA S2 Overview of significant tissue differences in microbiome composition based on relative abundances of bacterial families, individual OTUs and community diversity and richness.

DATA S3 Similarity percentage (SIMPER) analysis of bacterial community composition associated with different tissues of *P. margaritifera*.

DATA S4 IndicSpecies analysis of indicator OTUs/bacterial taxa associated with *P. margaritifera* tissues.

DATA S5 Overview of weighted Nearest Sequenced Taxon Index (NSTI) scores over all oyster samples.

DATA S6 PICRUSt and LEfSe analyses, an overview of differentially abundant KEGG pathways identified in the microbiomes of particular host tissues (per sample data provided).
