# Supplementary figures and images for: Microbiome of the Black-Lipped Pearl Oyster Pinctada margaritifera, a Multi-Tissue Description With Functional Profiling
Source: Front Microbiol. 2019 Jul 5;10:1548. doi: 10.3389/fmicb.2019.01548 (PMC6624473; doi:10.3389/fmicb.2019.01548)

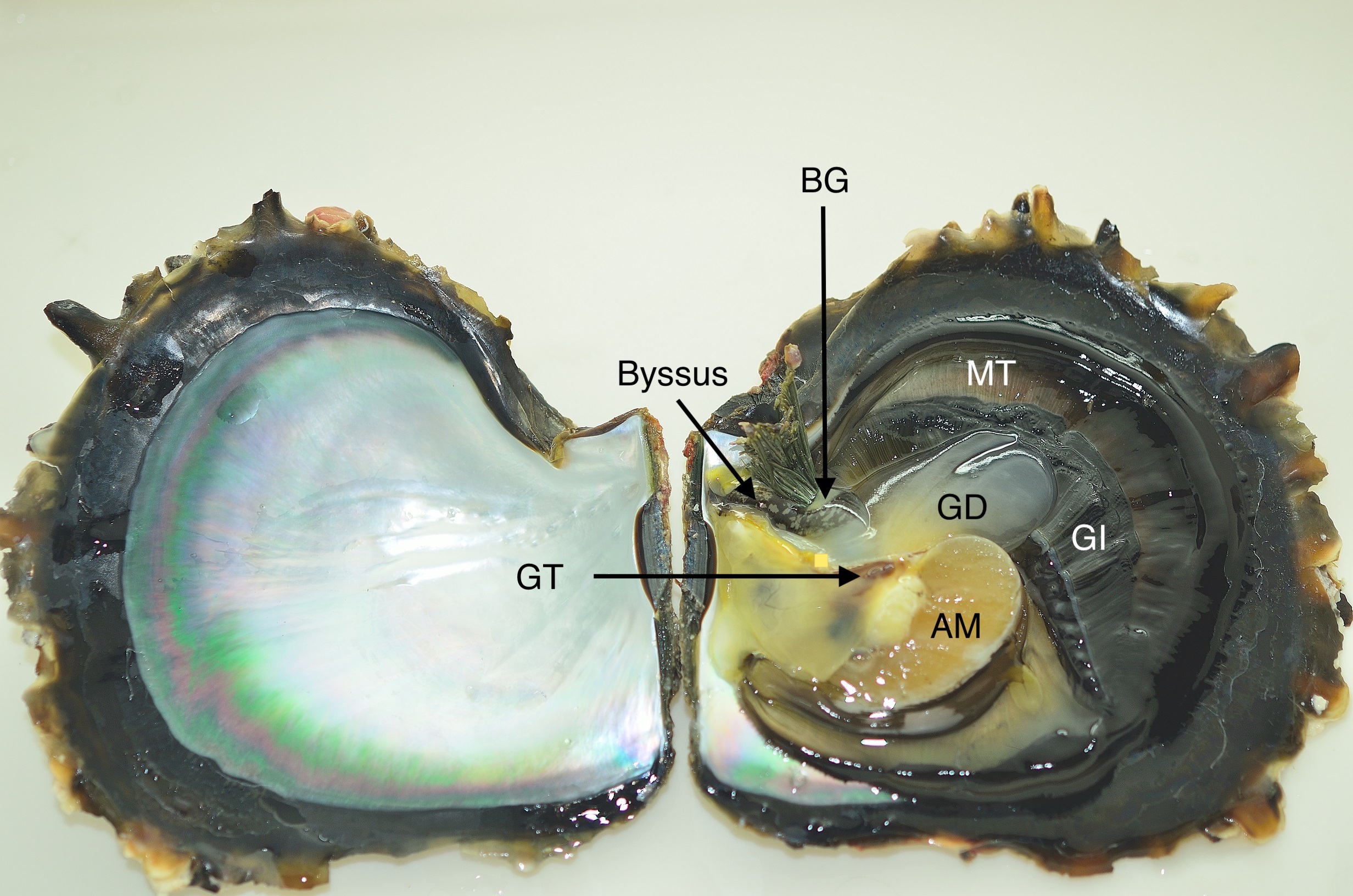

Supplement: FIGURE S1 — P. margaritifera compartments/tissues. Photo of each of the two shell valves showing, on the right, the collected tissues: GI, the gills; GD, the gonad; BG, the byssus gland; MT, the mantle; AM, the adductor muscle; and GT, the gut (shown partially on the photo). The haemolymph was collected from the byssus using a sterile syringe, while the mucus was collected on the mantle using a sterile swap. [file Image_1.JPEG]

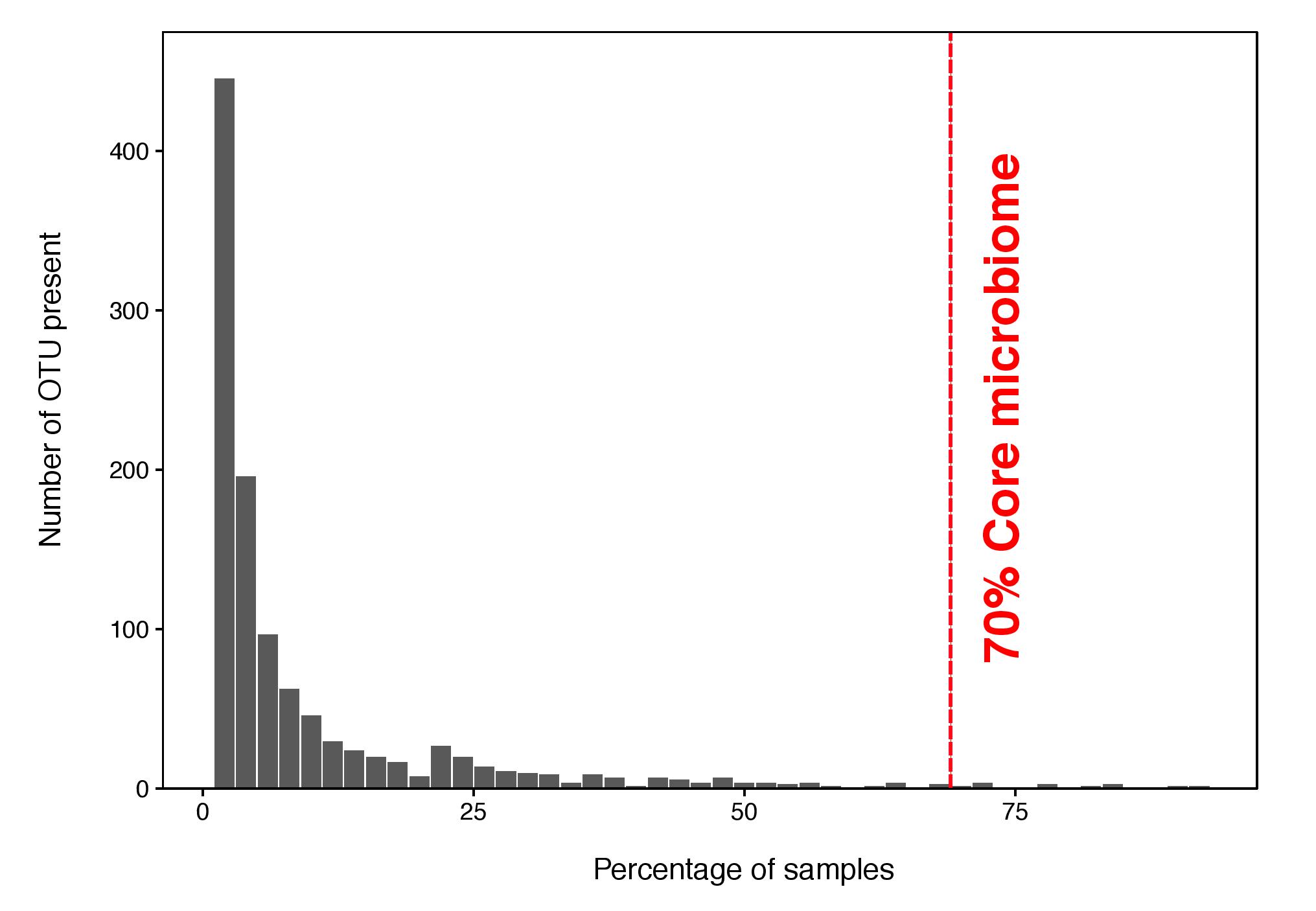

Supplement: FIGURE S2 — Plotted bacterial OTU abundance in core microbiome’s of 0 to 100% of samples of the pearl oyster P. margaritifera. [file Image_2.JPEG]
